# Idiopathic subglottic stenosis: an epidemiological single-center study

**DOI:** 10.1007/s00405-017-4512-0

**Published:** 2017-02-27

**Authors:** Maria Tyse Aarnæs, Leiv Sandvik, Kjell Brøndbo

**Affiliations:** 1 Department of Otorhinolaryngology/Head and Neck Surgery, Oslo University Hospital, Rikshospitalet, Mailboks 4956, 0424 Oslo, Norway; 2 Department of Biostatistics, Oslo University Hospital, Oslo, Norway

**Keywords:** Idiopathic, Subglottic, Stenosis, Mitomycin C

## Abstract

A retrospective epidemiological study of patients treated for idiopathic subglottic stenosis (ISS) during 2003–2013 at Oslo University Hospital, Rikshospitalet, was undertaken to assess its incidence, management and treatment outcomes. Out of a total of 123 patients with subglottic stenosis (84 female, 39 men), 38 patients were diagnosed with ISS, all of whom were female. Of these, 23 lived in the South-Eastern Norway Regional Health Authority, representing an incidence of 0.2 per 100,000 (95% CI 0.13–0.3) in this region of 2.9 million inhabitants. Mean age at diagnosis was 54 years (range 20–85 years), and the mean interval between symptom onset and diagnosis was 3.1 years. The 38 patients with ISS underwent a total of 132 operations between 2003 and 2013. All patients were managed endoscopically using laser surgery, with or without corticosteroids and Mitomycin C, with dilatation by balloon or bougie. Eight patients (21.1%) required only one procedure, while 30 patients (78.9%) had multiple operations. The median follow-up for all patients from the first operation was 5.3 years. The mean interval between procedures was 1 year for patients aged 20–48 years, 1.3 years for patients aged 49–61 years and 3.0 years for patients aged 62–85 years. No clinically significant complications were observed. In conclusion, the symptoms of ISS can be treated effectively with laser surgery and dilatation but the recurrence rate remains high and the time interval between operations does not increase with time, making ISS a continuing challenge.

## Introduction

Idiopathic subglottic stenosis (ISS) is a slow progressive inflammatory process of unknown aetiology which causes dyspnoea and stridor in adults. It is often misdiagnosed as adult-onset asthma and occurs predominantly in females [1–4]. A diagnosis of ISS is made when all other causes of subglottic stenosis, such as intubation-related airway stenosis, granulomatosis with polyangitis, bilateral vocal cord immobility and cancer, have been excluded [2, 5–8]. ISS is challenging to manage because the recurrence rate is high regardless of what type of surgical intervention is used.

The aim of the present study was to estimate the incidence of ISS in southern Norway, and to describe the ISS population at our center in terms of time to onset, symptoms, treatment and the need for reoperation.

## Materials and methods

The South-Eastern Norwegian Regional Health Authority is one of four health regions in Norway. Oslo University Hospital, Rikshospitalet is the only tertiary referral hospital in the region. Additionally, patients with subglottic stenosis in two northern regions are referred to the center due to a lack of local expertise. The incidence analysis included only those patients living in South-Eastern Norwegian Regional Health Authority, the population of which is 2.9 million.

All patients treated for subglottic stenosis at Oslo University Hospital, Rikshospitalet, during 2003–2013 were identified and their medical records were reviewed. A diagnosis of ISS was made if all other causes of stenosis were excluded, including intubation-related airway stenosis, sarcoidosis, granulomatosis with polyangitis, bilateral vocal cord immobility, and cancer and/or radiation in the larynx area. The following data were collected: age at symptom presentation, diagnosis and first operation; symptoms; dates of surgical interventions; the total number of surgical procedures; the type of surgical procedure; concomitant diseases (pulmonary disease, gastroesophageal reflux disease, hypertension, diabetes mellitus and obstructive sleep apnoea); nicotine consumption; and the presence of antineutrophil cytoplasmic antibodies (ANCA), to rule out granulomatosis with polyangitis. Patients were categorized into three age groups: Group 1, 28–48 years; Group 2, 49–61 years; and Group 3, 62–85 years and into ANCA- negative and ANCA- unknown groups.

Patients were treated endoscopically in preference to open surgery, because it is a minimally invasive approach with a low risk of complications and can usually be performed on an outpatient basis. Surgical procedures were carried out under general anaesthesia using jet ventilation. Generally, four incisions were made in the circumferential stenosis using a Sharplan 1030 laser attached to a Leica microscope. The typical laser settings were regular pulse, continuous mode and strength 1–2 W. In most patients, a soaked cotton pledget of Mitomycin C at a concentration of 2 mg/ml was applied twice for one minute per application, but during the early part of the study period, an intralesional corticosteroid injection was given instead. Dilatation was then carried out, using bougie or balloon dilatation until February 2011 and only balloon dilatation thereafter. The center has no formal post-operative follow-up regimen, but patients are requested to contact the center when symptoms recur.

The Privacy and Data Protection Office, CEO Executive Staff, Oslo University Hospital approved the study and data collection was authorized by the Norwegian Data Protection Authority.

### Statistical analysis

Data were analysed using the software package IBM SPSS 18 (SPSS Inc, Chicago, IL, US) with the level of significance set at *p* < 0.05. Quantitative summaries are given for patients requiring multiple procedures. Kaplan–Meier survival analysis was used to estimate the median time between reoperations. Log-rank statistics and Cox proportional hazards ratios were used to identify variables associated with the need for reoperation. When comparing mean time interval between procedures in two separate groups, the independent samples t-test was used to calculate p-values.

## Results

Over the study period, a total of 123 patients (84 females and 39 males) presented with subglottic stenosis. Of these, 38 patients were diagnosed as having ISS, all of whom were females. Twenty-three of the patients with ISS lived within the South-Eastern Norway Regional Health Authority. The incidence of ISS in the South-Eastern Norway Regional Health Authority was thus calculated to be 0.2 per 100,000 (95% confidence interval [CI] 0.13–0.3).

The mean age at diagnosis among all 38 patients was 54 years (range 20–85 years). There were 13 patients in Group 1 (28–48 years), 13 patients in Group 2 (49–61 years), and 12 patients in Group 3 (62–85 years). The mean time between symptom onset and diagnosis was 3.1 years. The median follow-up for all patients from the first operation was 5.3 years.

All patients presented with stridor and dyspnoea. Twenty-five patients (66%) had no other disease, six patients (16%) had both hypertension and diabetes mellitus and four patients (11%) had a history of gastroesophageal reflux. Twenty-five patients (66%) had negative serum markers for ANCA; ANCA testing was not performed in a further 13 patients (34%) but their medical records did not indicate suspicion of rheumatologic disease. Thirty-two patients (84%) reported no history of nicotine use.

There was no significant difference in total number of operations between the ANCA-negative and the ANCA-unknown. Mean age at diagnosis was 48 years for ANCA-negative and 65 years for ANCA-unknown, a significant difference (*p* = 0.001). Median follow-up time was 5 year for ANCA-negative and 9 years for ANCA-unknown, a significant difference (*p* = 0.016).

During 2003–2013, this cohort of 38 patients underwent 132 surgical procedures for ISS. All patients were treated endoscopically with laser surgery, with or without corticosteroids and Mitomycin C, and with dilatation by balloon or bougie. No patient required open surgery, and no clinically significant complications were observed. Eight patients (21%) underwent only one procedure, while the remaining 30 patients (79%) required multiple operations. These eight patients were ANCA-negative with a mean age of 53 years. Four received Mitomycin C and four did not. All were treated with laser and balloon dilatation, and they had a median follow-up time of 1.5 years. The median follow-up for all patients from the first operation was 5.3 years, with the longest follow-up being 10 years. Two patients had their first operation in 2013 and therefore had no more than 1 year of follow-up. The median time to reoperation was 1 year for Group 1, 1.3 years for Group 2 and 3 years for Group 3. Age at diagnosis had a significant influence on the interval between reoperations (*p* = 0.034), but in general, the interval between operations did not increase over time. Mitomycin C did not influence the time interval between operations.

## Discussion

To the best of our knowledge, this is the first study to assess the incidence of ISS. The findings confirm the rarity of this condition, estimated here to be only 0.2 per 100,000. It is reasonable to assume, however, that a number of cases remain undiagnosed. Patients with less serious disease who are diagnosed by pulmonologists at local hospitals may not have been referred to our center, and were thus not included. Delays in diagnosis of ISS are common: the majority of patients are first given a diagnosis of asthma and may be treated with inhalation steroids for a long time before being referred to an ear, nose and throat department [2, 9, 10]. Flexible laryngotracheoscopy is required to make the correct diagnosis at an early stage. Other than this caveat, the incidence data are likely to be relatively robust. Oslo University Hospital, Rikshospitalet is the only tertiary referral hospital for this health region, and thus performs all operations for ISS. Norwegian citizens have a unique identification number, which simplifies use of national registry systems, facilitating patient identification in different medical registries or when moving between regions or changing names. Moreover, since medical care is free for all Norwegian citizens and foreigners working in Norway, all patients are expected to seek medical attention when needed and selection bias due to socioeconomic status is of minor significance.

As in other studies [1–3, 8, 11–14], this study demonstrated homogeneity among ISS patients, who were mainly healthy females of European descent, diagnosed in their 50 s and requiring multiple procedures. The pathophysiology of the condition is unclear, but the age at diagnosis of approximately 50 years coincides with onset of menopause, supporting the hypothesis that hormones influence the etiologic process [1, 4, 11, 15]. It has been postulated that estrogen and progesterone influence the development of ISS, and a recent study has shown an imbalance between the estrogen receptors in patients with ISS. This imbalance leads to an inappropriate inflammatory response [16]. Blumin et al. postulated that extraesophageal reflux may cause ISS [8, 17–19]. In our study, four patients (11%) had gastroesophageal reflux disease, but it did not aggravate stenosis or induce more frequent surgical procedures. The rarity of ISS and its absence in men make a direct association with extraesophageal reflux unlikely.

Eight patients (21.1%) required only one procedure. There no distinguishing features in this group compared to those who underwent multiple surgeries, and some of these patients were included late in the study period and are likely to have further procedures in the future, so it seems unlikely that they differ meaningfully from the overall cohort. For the first time, we have shown that age has a significant influence on the time between reoperations. Whether this is a genuine finding linked to the pathology of the disease, or due to higher health expectations among younger patients, is unclear. Mitomycin C, an antimetabolite derived from *Streptomyces caespitosus*, has antineoplastic properties and has been shown to modulate the wound healing response by inhibiting fibroblast proliferation [20–25]. A number of human and animal studies have assessed the effect of topical Mitomycin C application, but its benefits remain controversial. In our study, Mitomycin C did not influence the need for additional operations or the time interval between operations, as has been reported elsewhere [2, 23–26], so we have ended use of this medication.

In spite of the majority of patients with ISS in Norway being treated at our hospital, the study is limited by the small number of patients. Biopsies from the lesions, as well as Cotton–Myer grading, are missing, making comparisons with other studies regarding these aspects impossible. Furthermore, the ANCA test was lacking in one-third of patients. However, categorized into ANCA-negative and ANCA-unknown, there was no significant difference in the total number of operations. The ANCA-unknown had a significant higher mean age and follow-up time, probably just a coincidence.

In conclusion, this retrospective study showed the annual incidence rate of ISS in South-Eastern Norway to be 0.2 per 100,000. Our findings indicate that ISS can be successfully treated using a minimally invasive endoscopic approach, effectively improving or even eliminating the symptoms of stridor. However, recurrence rate remains high and the interval between operations does not increase over time. Management of ISS thus remains a challenge. Multicentre studies are required to evaluate all aspects of the disease.
